# Increase of Insanity in the State of New York

**Published:** 1887-02

**Authors:** 


Increase of Insanity in the State of New York.—There were, on Oct. 1, 1886, in the various asylums of the State, 13,533 patients against 12,707 at the corresponding period of 1885, showing an increase of 826 during the year.

---

A French journal reports the case of a medical student who contracted gonorrhœa by sexual intercourse with a woman "*a la bouche.*" Such nastiness should be punished by a more severe disease.

---

*L'Abeille Medicale* offers a Waterbury watch to each new subscriber for 1887. Only wealthy journals can afford to be so generous to their patrons.

A Russian journal reports a case of syphilitic infection from a razor in a barber shop.

---

One of our homœopathic exchanges speaks of the furnace in the Buffalo *creamatory*.

